# Using e-modules for acquisition of complex diabetes skills in diabetes care providers in Rwanda

**DOI:** 10.1371/journal.pgph.0001638

**Published:** 2024-01-08

**Authors:** Melinda Chen, Simon-Pierre Niyonsenga, Edson Rwagasore, Elizabeth Lyden, Jessica Dudzinski, Cynthia Wilson, Shirley Delair, Florent Rutagarama

**Affiliations:** 1 Department of Pediatrics, Section of Pediatric Endocrinology, American Family Children’s Hospital, University of Wisconsin-Madison School of Medicine and Public Health, Madison, Wisconsin, United States of America; 2 Division of Non-Communicable Diseases, Rwanda Biomedical Center, Ministry of Health, Kigali, Rwanda; 3 Division of Public Health Surveillance and Emergency Preparedness and Response, Rwanda Biomedical Center, Ministry of Health, Kigali, Rwanda; 4 Department of Biostatistics, University of Nebraska Medical Center, Omaha, Nebraska, United States of America; 5 Independent, Overland Park, Kansas, United States of America; 6 Children’s Hospital and Medical Center, Omaha, Nebraska, United States of America; 7 Department of Pediatrics, Section of Pediatric Infectious Disease, University of Nebraska Medical Center, Omaha, Nebraska, United States of America; 8 Department of Pediatrics, Rwanda Military Hospital, Kigali, Rwanda; PLOS: Public Library of Science, UNITED STATES

## Abstract

Type 1 Diabetes (T1D) is life-threatening without appropriate treatment. Though pediatric endocrinology care is limited in Rwanda, a decentralized health system allows access to local non-communicable disease (NCD) nurses through a network of 42 district hospitals. Recent rapid expansion of internet access in the country makes virtual diabetes education initiatives possible. We investigated whether Rwandan NCD nurses receiving diabetes education via online e-modules could make similar educational gains in insulin adjustment skills (IAS) compared to NCD nurses educated in a conference-style setting, and whether they would maintain equivalent competency at 1 year after education. We randomized 21 district hospitals and their NCD nurses to participate in a 1.5-day educational conference centered around care of type 1 diabetes (Group 1), while nurses from the remaining 21 hospitals (Group 2) received accommodation and access to equivalent educational materials in e-module form. Both groups were requested to review initial course materials at 4, 8, and 12 months. Ten-point IAS assessments were administered before and after education or review at each time point. Groups 1 and 2 had equal improvement after education (+2.0 vs. +2.0, p = 0.47) and equal final score at baseline (6.0 vs. 6.0, p = 0.74). However, both groups showed a diminishing improvement over time, so that any gains were lost by 4 months in Group 1 and 8 months in Group 2. Group 1 showed greater attrition in participation over time (19% vs 58% continued participation at one year, p = 0.002). Groups did not differ in subjective confidence in IAS after education. Both groups identified existing or potential access barriers to their respective educational method. While further modifications should be trialed to ensure equitable access and to maintain long-term engagement, online education is a feasible method to teach complex subspecialty skills to providers working in low-resource settings.

## Introduction

Type 1 Diabetes (T1D) is an autoimmune disease characterized by a state of absolute low insulin production that can quickly lead to life-threatening diabetic ketoacidosis if insulin needs are not met appropriately. The prevalence of T1D in Africa is currently increasing due to a combination of increasing incidence and increased recognition on the part of healthcare providers, with subsequent appropriate management leading to greater long-term survival past diagnosis [1]. Rwanda is a small (26,338 km2), landlocked East African country, with an estimated 2000 patients with type 1 diabetes as of 2019 [2, 3]. The number of patients with type 1 diabetes receiving care in Rwanda has been steadily increasing for several years [2]. Non-profit organization Life for a Child has previously cared for over 600 of these via distribution of medication, supplies, and education [4] and continues to be active as a supplier. However, in recent years Rwanda has increased government-level support to sustain the continuum of care for T1D via decentralization of diabetes care to more accessible district-level hospitals [2]. This is further supported by in-country organizations such as the Rwanda Diabetes Association (RDA) and associated philanthropic donations, but overall leaves rural patients most likely to access local district hospitals for routine care, depending on non-communicable disease (NCD) nurses to care for a complex condition typically managed by subspecialists with specific training.

Care for patients with diabetes is complex, and in Rwanda is further complicated by supply access. Patients may have limited test strips and use older insulin formulations with less convenient pharmacokinetic profiles. Efficient use of limited supplies is possible [5], but providers must have a strong working knowledge of diabetes care to troubleshoot barriers and optimize patient care. Continuing medical education of health care providers in low resource settings is difficult both financially and logistically. Limited expertise, especially on a subspecialty level, makes continuing education difficult to deliver, and healthcare providers may lack the time and funding to access conferences or educational opportunities if they are available. On the other hand, Rwanda has enjoyed rapid introduction of cellular and internet access, such that 100% of a survey of 581 doctors reported daily internet use, with most doctors reporting using daily internet use to search for medical resources [6]. This could be a powerful tool for NCD nurses not only to collaborate and share information with each other, but to seek up-to-date and evidence-based information to assist in patient care.

This level of access and familiarity opens unique opportunities for continuing education, which are currently underutilized in Rwanda [6]. A study in Vietnam took advantage of widely available cellular phones to provide SMS-based continuing medical education to health care providers, focusing on HIV care. Over two iterations of their educational interventions, they found that module-based remote education facilitated improved knowledge acquisition based on the ability to build a broader foundation of knowledge over sequential days rather than sporadic teaching points [7]. However, data collection ceased after the participants’ final test, and therefore the longitudinal knowledge retention that would allow for long-term impact on patient care was not assessed. Another study based in South Africa demonstrated the potential acceptability of online education, though it was generally better accepted among younger, more computer literate individuals [8]. In addition, this study examined acceptability only, and did not attempt to examine efficacy of online education.

We sought to examine whether online education could be a reasonable platform to teach complex subspecialty skills in low-resource settings. We hypothesized that Rwandan NCD nurses receiving diabetes education via online e-modules could make similar educational gains in insulin adjustment skills (IAS) compared to NCD nurses educated in a conference-style setting, and that they would maintain equivalent competency at 1 year after education.

## Methods

### Ethics

This study was approved by the Institutional Review Board at the University of Nebraska Medical Center (IRB # 641-18-EX) and by the Republic of Rwanda National Ethics Committee (#455/RNEC/2018). Written informed consent was obtained from all participants in English or French based on participant preference prior to initiation of the conference or access to online modules. All NCD nurses traveling to the conference or invited to online participation were given access to educational materials for learning with a certificate of completion provided upon completion of education, regardless of participation in the study itself.

### Study procedures

In order to offer study inclusion to all NCD nurses in the country, all forty-two Rwandan district hospitals were randomized into two groups of 21 hospitals to receive either in-person or online education at baseline. Providers at Group 1 hospitals were invited to attend an in-person conference on the pathophysiology and care of type 1 diabetes, lasting 1.5 days in Kigali, Rwanda. Travel, lodging, and food were covered in order to minimize barriers to participation. Real-time translation of lectures was provided for all lectures. Providers at Group 2 hospitals received online access via an existing educational platform (Canvas) to all lecture slides from the conference in English and French, as well as recorded conference lectures in English, so that all providers would have access to materials in a language in which they had achieved fluency. Though they did not receive face to face learning time as the conference group did, they were encouraged to review lecture slides as many times as they felt necessary and at their own pace, and protected time at work was negotiated for them.

Education topics provided a broad overview of all aspects of short- and long-term care of type 1 diabetes (Table 1). Three to four hours of in-person teaching for Group 1 participants were devoted to reviewing the principles and practice of insulin dose adjustments, while Group 2 participants were encouraged to review these particular slides thoroughly with their protected work time. All topics were formed and reviewed to ensure accommodation of locally available resources. For example, insulin adjustment teaching focused primarily on the regular and intermediate acting insulins most accessible to Rwandan patients, while discussion of diabetic ketoacidosis incorporated situations with limited IV access, fluids, and electrolyte replacement.

**Table 1:** Summary of educational topics reviewed at the in-person conference.

Teaching Topics
Type 1 diabetes pathophysiology and epidemiology
Practical clinical care of type 1 diabetes for generalists
Insulin adjustments (interactive) with regular, intermediate insulins
Insulin adjustments with less common insulins–rapid, long-acting insulins
Dietary assessment and counseling
Long-term complications of type 1 diabetes
Management of diabetic ketoacidosis

**Fig 1:**
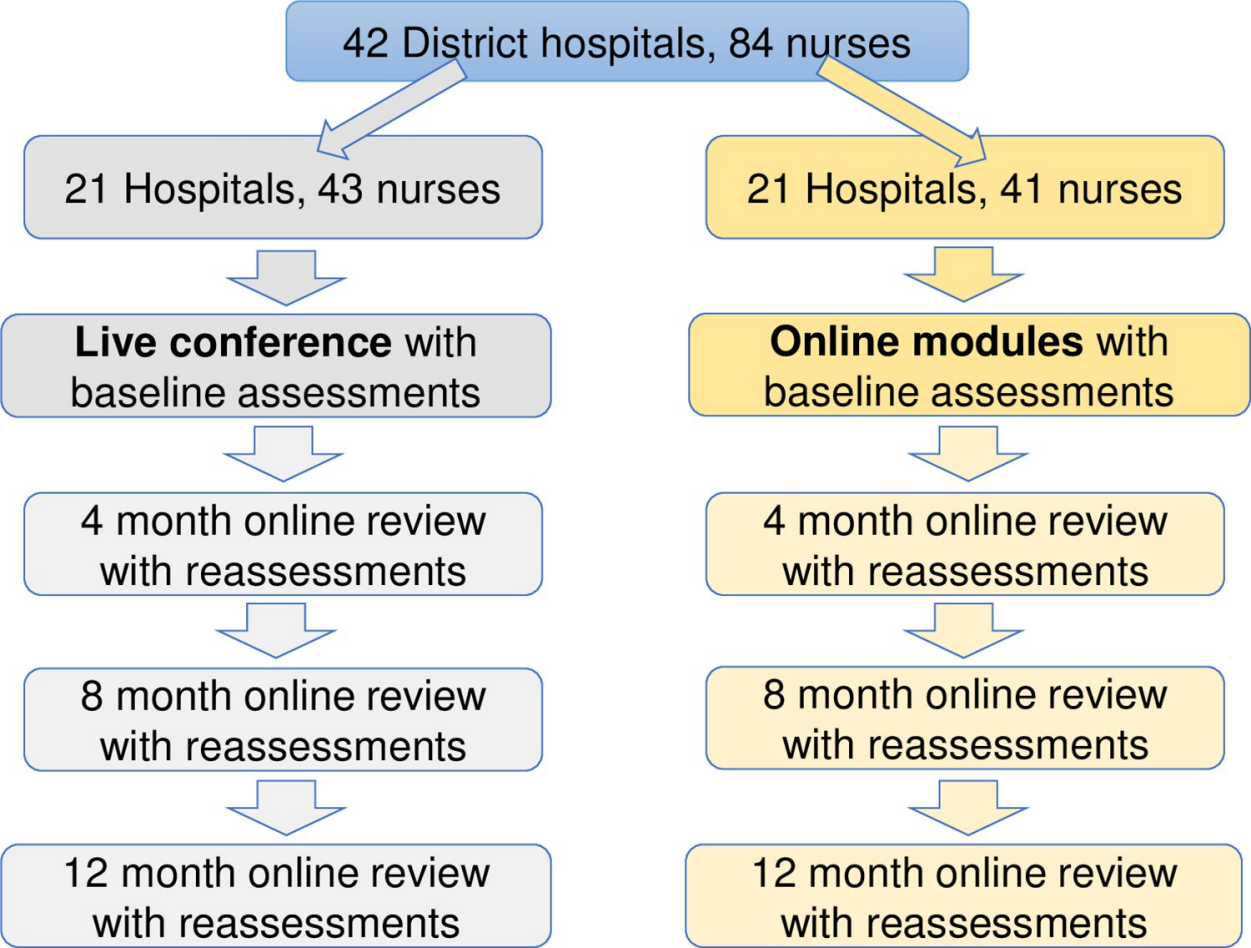
Education and assessment outline. Group 1 and Group 2 had identical online review materials, follow-up reminders, and assessments after baseline education.

At 4, 8, and 12 months after the initial education, both groups were invited to log into Canvas to review necessary lecture topics in order to allow participants opportunity to engage in self-motivated lifelong learning behaviors that would promote long-term retention of knowledge (Fig 1). A generous data plan was provided to both groups to meet their anticipated data needs to complete study activities.

Before and after each education and review timepoint (baseline, 4, 8, 12 months) participants of both groups were asked to complete a 10 question multiple choice quiz assessing their ability to make reasonable insulin dose adjustments. Each question was worth one point for a total of 10 points per quiz. Assessments were composed of questions randomized from a 40-question bank (S1 Appendix), for a total of 4 multiple choice assessments throughout the study with no repeated questions between assessments. Questions were relevant to the low-resource setting, encompassing locally available insulins, frequently missing blood sugar tests (common due to limited test strip availability), and integrating possible patient scenarios such as food insecurity, exercise, unstable home situations, and inability to appropriately store insulin, among others.

All participants were additionally asked to complete surveys based on Likert scales assessing the feasibility of each intervention. Group 1 was asked to assess the difficulty of accessing a similar conference in the future, assuming that they would be responsible for all costs of travel and lodging. Group 2 was asked to assess protected time, adequacy of provided data, and appropriateness of existing internet speed for their education needs. Both groups also completed surveys assessing perceived importance of the educational content as an addition to their existing knowledge base.

### Statistics

Initial recruitment goals were based on an anticipated 20% (0.20) improvement in retention of insulin adjustment knowledge over the 12-month period in both groups. We calculated that 80 participants with a 20% drop out rate would leave 64 participants to evaluate at 12 months. A sample size of 64 would produce a two-sided 95% confidence interval with a width equal to 0.21 when the sample proportion of improvement in retention from baseline to one year was 0.20. The lower limit of the confidence interval is 0.11 and the upper limit is 0.32.

Demographic variables (gender, location, and role) were expressed as proportion of the treatment group, time and patient care experience were expressed as mean ± SD, and performance and feasibility scores were summarized with medians and range. All baseline demographic data were included even if participants chose not to answer all demographic questions to maximize representation for each question. Comparisons of demographic variables between the groups, such as age, gender, education, urban vs. rural settings and health care provider roles in their home institution, were analyzed by Fisher’s exact test or the Mann-Whitney test as appropriate to the data. Spearman correlation coefficients were used to assess the correlation of scores with time and years of education.

All data were analyzed following the “intention to treat” paradigm, in which participants are analyzed according to their group assignment. Our primary outcome was the improvement of insulin adjustment performance at 12 months from baseline, measured on a 10-point scale. The Wilcoxon signed rank test was employed to compare pre-intervention baseline and 12-month assessment scores as well as assessment scores at each time point using post-review scores as the comparison to the pre-intervention baseline. The secondary outcome was the difference in insulin adjustment performance at 12 months between group 1 and group 2, using the Mann-Whitney test. A p<0.05 was considered significant.

Provider satisfaction was measured using Likert scales and reported descriptively. Feasibility of the training e-module platform was determined by evaluating the completion rate of pre- and post-assessments (which require intervening completion of modules) for all participants. The Mann-Whitney test was used to evaluate group differences in satisfaction survey responses as well as insulin adjustment scores at baseline and 12 months according to practitioner role, location of practice, and educational mode.

## Results

Mean time ± SD from baseline to 4-month follow up was 139.3 ± 12.9 days for Group 1 versus 77.9 ± 16.4 days for Group 2. Time from baseline to 8-month follow up was 268.8 ± 16.7 days for Group 1 and 208.7 ± 16.2 days for Group 2, while time from baseline to 12-month follow-up was 360.4 ± 18.9 days for Group 1 and 288.5 ± 10.5 days for Group 2 (p<0.001 between groups for all time intervals).

### Participant characteristics

Forty-three NCD nurses from 21 hospitals were invited, consented to participate in the study, and attended a 1.5-day conference, with 37 participants completing both pre- and post- assessments. Forty-one NCD nurses from 21 hospitals were invited to access online conference materials. Thirty-four responded to the initial invitation and consented to participate in the study, with 24 completing pre- and post-assessments.

Of those completing initial demographic surveys, no significant differences in terms of gender, age, time in practice, time in the NCD role, primary location of practice, or proportion involved in the Rwanda Diabetes Association were noted between group 1 and group 2 (Table 2). Across the entire study population, 69.2% of providers described their practice as based in a ‘rural area’.

**Table 2:** Demographic characteristics of Group 1 (in-person education) and Group 2 (e-module education) participants.

	Group 1/Conference	Group 2/E-module	p =
**Gender** N(%)			
Male	19 (50)	6 (40)	0.56
Female	19 (50)	9 (60)	
**RDA staff** N(%)			
Yes	3 (8)	4 (29)	0.07
No	35 (92)	10 (71)	
**Practice Location** N(%)			
City	4 (11)	1 (7)	0.79
Town	7 (18)	4 (19)	
Rural	27 (71)	9 (64)	
**T1D patients seen per month** Mean ± SD	39.5 ± 51.3	27.4 ± 26.5	0.33
**Time in medicine (years)** Mean ± SD	9.0 ± 6.2	8.4 ± 4.4	0.97
**Time in NCD role (years)** Mean ± SD	2.7 ± 1.6	3.1 ± 1.0	0.11

### Efficacy

At baseline, median (range) baseline knowledge was slightly lower in Group 1 (4.0 (0–7) vs Group 2, 4.5 (0–9), p = 0.02, Table 3). However, the two groups showed no significant differences in terms of improvement after education at baseline (Group 1 median (range) pre-education to post-education change +2.0 (-3-7) vs Group 2 +2.0 (-5-9), p = 0.47) (Table 4), or final score at baseline (Group 1 6.0 (1–8) vs Group 2 6.0 (1–9), p = 0.74). Improvement after education was associated with years of experience in medicine: individuals with greater experience in medicine showed greater improvement after education (R = 0.29, p = 0.049). No other demographic factor was associated with improvement upon education at baseline (Table 5).

**Table 3:** Median test scores at baseline and after education/review at 4, 8, and 12 months.

Table 2	Baseline score (before education)		Scores after education/review											
Education mode	Baseline		Baseline education			4-month review			8-month review			12-month review		
	N		N		p*	N		p	N		p	N		p
Group 1 In-person	43	4.0 (0–7)	37	6.0 (1–8)	<0.0001	13	5.0 (2–7)	0.08	6	6.0 (4–9)	0.19	7	4.0 (2–6)	0.62
Group 2 E-module	34	4.5 (0–9)	24	6.0 (1–9)	0.02	23	5.0 (3–9)	0.03	14	5.0 (2–7)	0.85	18	5.0 (3–7)	0.39

* p values denote difference in scores compared to pre-education baseline.

**Table 4:** Median difference in test scores compared to pre-education baseline by group.

Table 3	Post-education change from pre-education knowledge at baseline											
Education mode	Baseline education			4-month review			8-month review			12-month review		
	N =	Post Score	p*	N =	Post Score	p	N =	Post Score	p	N =	Post Score	p
Group 1 In-person	37	2.0 (-3-7)	0.47	13	1.0 (-5-5)	0.83	6	2.5 (-1-5)	0.16	7	0.0 (-3-4)	0.64
Group 2 E-module	24	2.0 (-5-9)		23	1.0 (-3-6)		14	0.0 (-4-5)		18	0.0 (-4-3)	

*p values denote score difference between groups at each time point.

**Table 5:** Association of participant demographic factors with change in IAS scores after initial education.

	Median IAS Score Improvement	p =
**Gender**		
Male	2.0 (-3-9)	0.86
Female	2.0 (-2-7)	
**Staff member with Rwanda Diabetes Association**		
Yes	2.0 (-2-3)	0.31
No	2.0 (-3-9)	
**Location of Primary Practice**		
City	1.0 (-1-4)	0.52
Town/Rural	2.0 (-3-9)	
	*R =*	p
**Type 1 diabetes patients seen**		
Per day	-0.22	0.16
Per week	0.05	0.74
Per month	0.02	0.92
**Time practicing medicine (years)**	0.29	**0.049**
**Time in NCD position (years)**	0.02	0.90

Fisher’s exact test, Kruskal-Wallis test, and Spearman correlation coefficients were used to correlate demographic characteristics with improvement in IAS after baseline education.

Neither pre-review or post-review scores differed between Groups 1 and 2 at 4-, 8-, or 12-month follow up.

### Durability

Group 1 showed poorer retention of IAS, with all educational benefit lost by 4 months compared to pre-intervention baseline, even after the opportunity to review education topics. Group 2 also showed reduced knowledge retention at 4 months, but continued to have significant improvement when compared to pre-education baseline. However, by 12 months neither group differed from its pre-education baseline (Table 3).

Groups did not differ in improvement with education or review (when compared to baseline scores) at any time point (Table 4). Due to participant dropout, we additionally compared groups at 12 months using last observation carried forward (LOCF) and found nearly identical results. Group 1 showed a median score of 0.0 (-3-5) and Group 2 showed a median score of 0.0 (-4-6) at 12 months (p = 0.65), indicating that durability results were not affected by dropout at 12 months.

### Feasibility

Groups did not differ in their confidence in IAS after education (Group 1 = 83% vs Group 2 = 100%, p = 0.17) or perceived applicability of education to their practice (Group 1 = 92% vs Group 2 = 100%, p = 0.55) (Table 6). However, Group 1 did feel more strongly at the end of their education that they understood the differences between type 1 and type 2 diabetes (44.4% strongly agreed and 52.8% agreed in Group 1, vs 8.3% strongly agree and 91.7% agree in Group 2, p = 0.04).

**Table 6:** Participant perspectives of education efficacy by group.

	Group 1 N(%)					Group 2 N(%)					p =
The education topics…	Strongly agree	Agree	Neutral	Disagree	Strongly Disagree	Strongly agree	Agree	Neutral	Disagree	Strongly Disagree	
• Were applicable to my patient practice.	15 (39.5)	20 (52.6)	-	-	3 (7.9)	2 (16.7)	10 (83.3)	-	-	-	0.17
• Contained new material I had not learned before.	4 (10.5)	20 (52.6)	2 (5.3)	10 (26.3)	2 (5.3)	-	4 (33.3)	1 (8.3)	7 (58.3)	-	0.26
• Contained subjects that were necessary for me to review.	16 (43.2)	13 (35.1)	1 (2.7)	5 (13.5)	2 (5.4)	-	9 (75)	1 (8.3)	2 (16.7)	-	**0.01**
I found it difficult to understand the education content because…	Strongly Agree	Agree	Neutral	Disagree	Strongly Disagree	Strongly agree	Agree	Neutral	Disagree	Strongly Disagree	
• Of language difficulties	-	5 (13.2)	1 (2.6)	26 (68.4)	6 (15.8)	-	3 (25)	1 (8.3)	6 (50)	2 (16.7)	0.43
• It was not well adapted for my practice setting	-	5 (13.5)	-	23 (62.2)	9 (24.3)	-	-	2 (16.7)	8 (66.7)	2 (16.7)	0.09
• The information was more complex than I was prepared for	1 (2.6)	3 (7.9)	1 (2.6)	26 (68.4)	7 (18.4)	-	3 (25)	1 (8.3)	8 (66.7)	-	0.18
I feel confident making insulin adjustments	8 (21.1)	23 (60.5)	1 (2.6)	4 (10.5)	2 (5.3)	4 (33.3)	8 (66.7)	-	-	-	0.75
I improved my knowledge of the differences between type 1 and type 2 diabetes	16 (44.4)	19 (52.8)	-	-	1 (2.8)	1 (8.3)	11 (91.7)	-	-	-	**0.04**
I am confident that I can recognize short term complications of type 1 diabetes	13 (34.2)	21 (55.3)	-	3 (7.9)	1 (2.6)	3 (25)	8 (66.7)	1 (8.3)	-	-	0.39
I am confident that I can assess for long term complications of type 1 diabetes	12 (31.6)	23 (60.5)	-	1 (2.6)	2 (5.3)	2 (18.2)	8 (72.7)	1 (9.1)	-	-	0.42

Some participants in Group 1 felt that requirements to fund their own lodging, travel, or registration fee would be barriers to accessing in-person education (18.4%, 29.7%, and 30.6%, respectively) (Table 7). Group 2 participants most frequently identified time commitment (83%) as a barrier to completing the requested activities. Almost as many (75%) identified difficulty accessing the platform or a slow internet connection as impeding their progress (Table 8).

**Table 7:** Group 1 participant perspectives on accessibility factors relevant to in-person education.

Accessibility–Group 1 N(%)	Strongly Agree	Agree	Neutral	Disagree	Strongly Disagree
It was difficult for me to make the time to come to the conference.	4 (10.5)	6 (15.8)	3 (7.9)	20 (52.6)	5 (13.2)
The funds to assist with travel were insufficient.	4 (11.4)	10 (28.6)	1 (2.9)	11 (31.4)	9 (25.7)
The conference length was too long.	1 (2.7)	2 (5.4)	1 (2.7)	21 (56.8)	12 (32.4)
How much would the following factors be a barrier to attending a similar conference in the future?	Extreme		Moderate	Somewhat	Not A Barrier
• Paying for own lodging	4 (10.5)		1 (2.6)	2 (5.3)	31 (81.6)
• Paying for own travel	4 (10.8)		4 (10.8)	3 (8.1)	26 (70.2)
• A small registration fee to offset cost of food	3 (8.3)		3 (8.3)	5 (13.9)	25 (69.4)

**Table 8:** Group 2 perspectives on accessibility factors relevant to online education.

Accessibility–Group 2 N(%)				
How much were the following factors a barrier to completing the online modules within the expected time frame?	Extreme	Moderate	Somewhat	Not A Barrier
• Technical difficulties accessing the online modules	-	4 (33.3)	5 (41.7)	3 (25)
• A slow connection making videos and recordings skip or difficult to understand	1 (8.3)	4 (33.3)	4 (33.3)	3 (25)
• Not having enough data to complete all modules	-	2 (16.7)	5 (41.7)	5 (41.7)
• Time commitment	-	6 (50)	4 (33.3)	2 (16.7)

Both groups had comparable success completing the full sequence of baseline pre-assessment, education, and post-assessment (37/43 or 86% of Group 1 and 24/34 or 70.6% of Group 2, p = 0.16). Both groups had attrition in participation after baseline (Table 3), though the trend between the 4- and 12-month follow up was for greater attrition in Group 1, who had initially received education in the in-person format. By 12 months, participation in Group 1 (7/37, 18.9%) was about one-third the participation of Group 2 (14 of 24, 58%) (p = 0.002).

## Discussion

In this study, online education was of similar efficacy compared to in-person education for complex skill acquisition at baseline. While a longer time in practice was associated with greater improvement, no other demographic factors were associated with better scores after education. This suggests that online education can teach complex cognitive skills to health care workers in a variety of low-resource practice settings, and can be applied to subspecialty education where available teachers are relatively scarce.

Unfortunately, despite effective skills acquisition on initial teaching, neither educational method showed evidence of transferring knowledge in a durable fashion, even with a schedule that encouraged and enabled periodic review with online materials. The loss of participation over time, particularly in Group 1, is a weakness that limits our understanding of the severity of IAS loss over time. However, we do believe that this dropout is unlikely to have affected our overall conclusions, as these results did not change even in a sensitivity analysis at our primary endpoint of 12 months, which carried forward each participant’s last score. We believe the marked attrition of participation and loss of IAS in remaining participants over a year of study represent the possibility of a general failure to continue engaging in the material over time. This may have contributed to the lack of durability, as repetition of knowledge contributes to its retention [9]. Other studies have noted low engagement with non-traditional learning methods [10, 11], even with frequent prompts to engage in available study materials [11]. Thus, while online learning seems to be a promising option for initial knowledge transfer, greater attention and study should be devoted specifically toward improving long-term engagement for purposes of educational reinforcement.

In future, retention of interest and education may require mixed learning methods. Online materials used for reinforcement of in-person teaching have been favorably received by learners. In a study of combined educational methods, authors noted that students consistently scored better on quizzes when allowed to collaborate in groups, and the use of gamification in the form of interactive quizzes allowed for low-pressure continued education. However, most students continued to employ traditional learning methods such as written materials to prepare in advance for the online gamified content [12]. This suggests that collaboration as well as access to both traditional and innovative methods is ideal in order to optimally meet students’ varying learning styles. Similarly, in the low-resource setting, an SMS-related educational study found that participants improved their knowledge the most when the SMS quizzes stimulated further reading and study using traditional methods [13, 14]. Thus, retaining our current lecture and teaching materials is important, but may be enhanced by additional features that allow the opportunity for peer collaboration or interactive learning.

In addition to mixed learning methods, staggered introduction of topics rather than a one-time educational session may be helpful in maintaining learner engagement. Our study provided comprehensive diabetes education for both groups. Online education allowed limited flexibility for self-paced learning, while this flexibility was not logistically feasible for conference-style education by Group 1. However, our intervention and online platform were not designed to explore this question, which deserves further exploration as the frequency and acceptability of online learning increases.

The differing length of time from baseline to follow up between Group 1 and Group 2 was largely attributable to unanticipated prolonged time becoming conversant with the online platform in Group 2. This led to relatively late initial education and assessment for many participants and subsequently shortened interval between baseline education and review time points. This shortened time may have contributed to the perceived longer duration of educational gains in Group 2. However, this additional time gaining competency in the online platform may also have allowed Group 2 participants greater ease in remaining engaged in the online format over time. A deeper and more dedicated introduction to navigating the online system may be helpful in future iterations, in settings where online learning is relatively new and uncommon.

Literature on teaching done entirely online continues to be scarce in the low-resource environment. Within higher-resource settings, continued engagement and participation in pure online learning is also a struggle. In a review of online distance nursing education methods, factors related to student retention include faculty responsiveness to needs and interaction with students [15]. In the future, more faculty involvement and availability for questions could be structured into online learning courses.

This study is the first, to our knowledge, that compares the efficacy and durability of online versus in-person educational methods for teaching subspecialty skills in the low-resource setting. Our study has several strengths including high interest and initial engagement, randomized hospital allocation to intervention group, and longitudinal follow up. However, we also acknowledge several weaknesses. Blinding to group was not possible due to the nature of intervention, though we did ensure that all nurses at a given hospital were in the same group so as to avoid cross-contamination between educational methods. Our assessments of insulin adjustment skills, though created and reviewed by specialists in diabetes care and education, were not validated quizzes as no such measures exist in the low-resource setting, to our knowledge. However, participants largely felt that the created content was relevant and applicable to their practice setting.

Despite wide and rapid expansion of the internet many participants in Group 2 continued to note poor connection as a barrier to completing required activities. Bandwidth capabilities should be considered in development of any future online education modules to ensure smooth access to class materials, and creative means will be required to do this while still allowing materials to be innovative and interactive. In addition, many participants noted that time was a barrier to completion of the required activities even when protected time had been negotiated. This suggests that within this setting, approaches toward online resources should be sensitive to the limited time available to working adults for independent learning.

Online education has potential to effectively teach complex skills to learners in low-resource settings. This is a potentially powerful tool in a high-need setting where finances and logistics are a constant barrier to bringing subspecialty care to patients. However, a vital barrier to overcome is difficulty maintaining interest and engagement over the long-term. Increasing opportunities for interaction and dedicated faculty involvement in this online platform may be crucial to its successful implementation as an educational tool.

## Supporting information

S1 Checklist Questionnaire on inclusivity in global research. (DOCX)

S1 Appendix Question bank used for all assessments. Forty questions were divided randomly into 4 separate assessments to be used as described at baseline and the 4, 8, and 12 month time points, so that no question was repeated between assessments.
(DOCX)

S1 Data De-identified data underlying study results in compliance with PLoS data policy. “Key” tab relates survey questions with the table identifier.
(XLSX)
