# Supplementary material for: Using e-modules for acquisition of complex diabetes skills in diabetes care providers in Rwanda
Source: PLOS Glob Public Health. 2024 Jan 8;4(1):e0001638. doi: 10.1371/journal.pgph.0001638 (PMC10773950; doi:10.1371/journal.pgph.0001638)
Supplement: S1 Appendix — Forty questions were divided randomly into 4 separate assessments to be used as described at baseline and the 4, 8, and 12 month time points, so that no question was repeated between assessments. (DOCX) [file pgph.0001638.s002.docx]

Problem 1:

An active 14 year old type 1 diabetic comes to clinic with his numbers. He is attending school regularly and enjoys playing football in the evenings with his friends. How can you advise him for a next step?

|  | **Breakfast** | **Lunch** | **Supper** | **Bedtime** |
| --- | --- | --- | --- | --- |
| Monday | 356 |  |  |  |
| Tuesday | 412 |  |  |  |
| Wednesday | 309 |  |  |  |
| Thursday | 282 |  |  |  |
| Friday | 374 |  |  |  |

1. He is showing dawn phenomenon - increase his evening NPH dose.
2. Increase all his insulin doses.
3. More information is needed - Ask him to check his blood sugar more often, 7-10 times every day for the next 2 weeks and return
4. More information is needed - Ask him to check a blood sugar 2-3 times per day, including an overnight check before making adjustments.

Problem 2

A 17 year comes to clinic. Her HbA1c is 11%. What is your next step?

Blood sugar log

8 AM – 212

10:30 AM – 191

9:30 AM – 316

7:45 AM – 248

8:12 AM – 112

1. She is showing Somogyi phenomenon – decrease her evening NPH dose.
2. All of her glucoses are high - increase all her insulin doses.
3. More information is needed - Ask her to check her blood sugar more often, 7-10 times every day for the next 2 weeks before making adjustments.
4. More information is needed - Ask her to check her blood sugar 3-4 times per day including an overnight check before making adjustments.

Problem 3

A 20 year old type 1 diabetic comes to clinic. His HbA1c is 9.2%. How can you advise him?

|  | **Breakfast** | **Lunch** | **Supper** | **Bedtime** |
| --- | --- | --- | --- | --- |
| Monday |  |  | 170 |  |
| Tuesday |  |  | 191 |  |
| Wednesday |  |  | 203 |  |
| Thursday |  |  | 149 |  |
| Friday |  |  | 217 |  |

1. Increase all his insulin doses because his blood sugars are always high.
2. Increase his morning regular insulin, check glucoses at mid-day and suppertime for a few days
3. Check glucoses at different times of day for a few days before making changes – it is best if glucoses are checked randomly
4. Check glucoses at different times of day for a few days before making changes – they will be most informative if they are checked before and after an insulin shot has taken effect

Problem 4:

A 25 year old student with type 1 diabetes returns for follow up with the following numbers. He works on Fridays, Saturdays, and Sundays as a laborer. What can you advise him about his blood sugars?

|  | **Breakfast** | **Lunch** | **Supper** | **Bedtime** | **Overnight** |
| --- | --- | --- | --- | --- | --- |
| Monday | 309 | 167 |  |  |  |
| Tuesday |  |  |  |  |  |
| Wednesday | 137 |  |  | 106 |  |
| Thursday |  |  | 153 |  |  |
| Friday |  |  |  |  | 67 |
| Saturday |  | 321 |  |  | 52 (woke up and felt low) |
| Sunday | 427 | 287 |  |  | (woke up and drank juice, did not check) |

1. His evening NPH is too high because he always goes low overnight
2. His evening NPH is too low because he is always high in the morning.
3. He may be having delayed hypoglycemia from his physical labor on the weekends.
4. There is no clear pattern and he should check blood sugars more often.

Problem 5

A 12 year old boy comes to clinic. His HbA1c is 8.2%. He is not missing any school, and he likes to play football with his friends after school has finished for the day before going home for dinner. He takes the following doses:

|  | Breakfast | Suppertime |
| --- | --- | --- |
| Regular | 6 | 4 |
| NPH | 10 | 8 |

What is your assessment?

|  | **Breakfast** | **Lunch** | **Supper** | **Bedtime** | **Overnight** |
| --- | --- | --- | --- | --- | --- |
| Monday | 137 |  |  | 67 |  |
| Tuesday | 312 |  |  |  |  |
| Wednesday |  |  | 221 | 54 |  |
| Thursday |  | 98 | 287 |  |  |
| Friday | 287 |  |  |  | 67 |
| Saturday |  |  |  | 146 |  |
| Sunday |  |  |  | 101 |  |

1. His evening regular insulin must be too high because of his frequent lows at bedtime
2. His evening NPH is too low because he is always high in the morning.
3. He may be having delayed hypoglycemia from playing football, and he should decrease his evening regular insulin if he is physically active.
4. There is no clear pattern and he should check blood sugars more often.

Problem 6:

A 7 year old boy with type 1 diabetes comes to clinic. His mother is frustrated that every time she checks his blood sugar, he seems high. He takes the following doses:

|  | Morning | Evening |
| --- | --- | --- |
| Regular | 2 | 1 |
| NPH | 6 | 2 |

What should your next step be?

|  | **Breakfast** | **Lunch** | **Supper** | **Bedtime** |
| --- | --- | --- | --- | --- |
| Monday |  |  |  | 167 |
| Tuesday | 412 |  |  |  |
| Wednesday |  |  | 145 |  |
| Thursday | 282 |  |  |  |
| Friday | 374 |  |  |  |
| Saturday |  | 112 |  |  |
| Sunday |  |  |  | 132 |

1. Explain that his high glucoses are due to dawn phenomenon and increase his evening NPH to 3 units
2. Explain that his high glucoses are due to improper insulin dosing at suppertime and increase his evening regular to 2 units
3. Ask his mother to check glucoses overnight for more information
4. Since over half his glucoses are close to target range, do not make any changes

Problem 7

A 12 year old type 1 diabetic comes into clinic. What would you tell him?

|  | **Breakfast** | **Lunch** | **Supper** | **Bedtime** |
| --- | --- | --- | --- | --- |
| Monday |  |  |  | 128 |
| Tuesday | 412 |  |  |  |
| Wednesday |  |  |  | 109 |
| Thursday | 282 |  |  |  |
| Friday | 374 |  |  |  |

1. He is clearly showing dawn phenomenon - increase his evening NPH dose.
2. He is clearly showing Somogyi phenomenon – decrease his evening NPH dose.
3. More information is needed - Ask him to check his blood sugar more often, 6-7 times every day
4. More information is needed - Ask him to check overnight for a few days before making insulin adjustments.

Problem 8:

A 15 year old type 1 diabetic comes in for follow up. He has noticed that whenever he stays with his uncle his blood sugars are always high. He says that the food in both homes is about the same, although his aunt uses much more palm oil. What would you advise him?

1. Since he is only at his uncle’s house once in a while, it is fine to do nothing differently.
2. Increase his regular insulin during meals at his uncle’s house:
3. Increase his NPH insulin during meals at his uncle’s house
4. Increase both his regular and NPH insulin during all meals regardless of location, to keep his insulin regimen simple

Problem 9:

An 8 year old new diabetic is ready to start insulin. Your plan is to start her on:

|  | Morning | Evening |
| --- | --- | --- |
| Regular | 2 | 1 |
| NPH | 4 | 2 |

The family is poor and live in a rural area. Her mother states that they usually do not eat breakfast and have a very large lunch, and have a very small dinner. How should you change your insulin plan?

1. 3 units of NPH in the morning, 1 units of regular insulin and 2 units of NPH in the evening.
2. 3 units of NPH in the morning, 1 unit of regular insulin and 3-4 units of NPH in the evening.
3. 0 units of regular and 5 units of NPH in the morning, 0.5 units of regular insulin and 2 units of NPH in the evening.
4. 3 units of NPH in the morning, 2 units of regular insulin at lunchtime, 0.5 unit of regular and 2 units of NPH in the evening.
5. Both C and D are reasonable options.

Problem 10

A 26 year old diabetic returns for follow up. At his last visit, you prescribed:

|  | Morning | Evening |
| --- | --- | --- |
| Regular | 10 | 5 |
| NPH | 20 | 12 |

Since his last visit, he has recently lost his job. To save money on food to help pay for his insulin, he usually skips breakfast, eats a large lunch and a smaller supper. He asks you if he still needs insulin because it is often making him feel low.

|  | **Breakfast** | **Lunch** | **Supper** | **Bedtime** |
| --- | --- | --- | --- | --- |
| Monday | 68 |  | 312 |  |
| Tuesday |  | 46 |  |  |
| Wednesday |  |  |  |  |
| Thursday |  |  |  | 72 |
| Friday |  |  |  |  |

What should you tell him next?

1. He can stop his insulin because he is having too many lows.
2. Decrease his NPH doses and tell him to take regular insulin only when he has food to eat.
3. He can stop his regular insulin and take NPH only to save money on insulin.
4. He could switch to 30 units of 70/30 at breakfast and 17 units of 70/30 at supper to save money on insulin.

Problem 11:

A 17 year old girl with type 1 diabetes returns to clinic. Her blood sugars were very good at her last appointment:

|  | **Breakfast** | **Lunch** | **Supper** | **Bedtime** |
| --- | --- | --- | --- | --- |
| Monday | 123 | 137 |  |  |
| Tuesday |  | 109 | 98 |  |
| Wednesday |  |  |  | 157 |
| Thursday | 143 |  |  |  |
| Friday |  |  |  |  |

She was staying at a relative’s house for a week, and her blood sugars started to become high while she was there and have continued to be high since she came back:

|  | **Breakfast** | **Lunch** | **Supper** | **Bedtime** |
| --- | --- | --- | --- | --- |
| Monday | 270 |  |  | 315 |
| Tuesday | 267 |  |  |  |
| Wednesday |  | 421 | 354 |  |
| Thursday |  |  |  |  |
| Friday |  |  |  |  |

What is the most appropriate next step?

1. Even though it is expensive, she should replace her insulin as her insulin may not have been stored correctly at her relative’s house.
2. Have a stern talk with her about taking her insulin doses as prescribed.
3. Increase all of her insulin doses.
4. Tell her that she should eat fewer carbohydrates.

Problem 12:

A 7 year old girl with type 1 diabetes is diagnosed with malaria. You give her a course of Coartem to take at home. What should you tell her about her diabetes care?

1. If she starts to have lower blood sugars at home she should stop taking her insulin until her medication is completed.
2. If she has ketones she should treat it with extra water and insulin. When her ketones turn negative she can stop checking her urine.
3. If her mother notices fast breathing it is probably the malaria and she should go to the pharmacy to try a different malaria medication since the Coartem is not working.
4. She should check her ketones occasionally throughout her illness. Fast breathing may be a sign of worsening malaria, but may also be a sign of ketones that need treatment with extra insulin.

Problem 13:

A 15 year old girl comes to clinic with a stomachache and rapid breathing. She has not been eating and drinking much because of appetite loss. She has large ketones in her urine and her malaria test is positive. What should you do next?

1. Give her a course of antimalarials and send her home.
2. Admit her for insulin drip therapy and IV hydration.
3. Check her blood sugar and give her more insulin than she would usually use to correct.
4. Check her blood sugar log to see if it looks like she has been skipping insulin doses.

Problem 14:

A 16 year old Muslim girl will be celebrating Ramadan with her family soon. They ask you how they should change their daughter’s insulin regimen.

1. Don’t take any insulin during the day since she will be fasting during the day
2. Decrease all her insulin doses – since her eating hours are limited, she will likely need less insulin
3. She cannot celebrate Ramadan because as a diabetic, she is required to eat regularly.
4. She should shift her regular insulin to the nighttime hours to cover her meals, and she should check her blood sugars to look for highs and lows while she is on a different schedule.

Problem 15

A 7 year old boy comes in with the following numbers:

|  | **Breakfast** | **Lunch** | **Supper** | **Bedtime** |
| --- | --- | --- | --- | --- |
| Monday | 119 | 137 |  |  |
| Tuesday | (drank a bottle of soda because he felt low) | 376 |  |  |
| Wednesday |  | 98 |  |  |
| Thursday | 57 | 421 |  |  |
| Friday |  |  | (did not take regular insulin because he felt low) | 476 |

What is the best next step?

1. Increase his regular insulin at breakfast time because he is often high at midday
2. Assess how he is treating his lows, as he may be overtreating himself
3. Decrease his evening NPH because he was high in the morning
4. Assess the timing of his meals – he may be high because his meals are not coinciding with the peak of his NPH

Problem 16:

A 21 year old diabetes patient comes in with the following numbers. He has breakfast at 9 AM, lunch at 12 PM, and supper at 5 PM. His doses are:

|  | 9 AM | 5 PM |
| --- | --- | --- |
| Regular | 8 | 5 |
| NPH | 16 | 10 |

What do you advise him about his insulin doses?

|  | **8 AM** | **12 PM** | **2 PM** | **5 PM** | **9 PM** |
| --- | --- | --- | --- | --- | --- |
| Monday | 137 |  |  | Felt low – ate supper |  |
| Tuesday |  | 181 |  | 45 |  |
| Wednesday | 98 |  |  |  |  |
| Thursday |  |  |  |  | 216 |
| Friday |  |  | 265 | 57 |  |

1. Decrease his morning NPH because he is frequent low at suppertime
2. Increase his morning NPH because his post-lunchtime blood sugar is higher than target
3. Decrease his suppertime regular insulin because his bedtime blood sugars are high
4. Advise him to eat lunch later so that the blood sugar peak from food coincides with the peak of his NPH.

Problem 17

A 5 year old diabetes patient comes in with her mother with the following numbers. She has breakfast at 8 AM, lunch at 12 PM, and supper when her father comes home from work at 8 PM. Her doses are:

|  | 8 AM | 6 PM |
| --- | --- | --- |
| Regular | 1 | 2 |
| NPH | 2.5 | 2 |

What do you advise her mother about her insulin doses?

|  | **Breakfast** | **Lunch** | **Supper (8 PM)** | **Bedtime (9 PM)** |
| --- | --- | --- | --- | --- |
| Monday | 137 |  | Felt low – ate supper |  |
| Tuesday |  | 151 | 45 |  |
| Wednesday | 85 |  |  |  |
| Thursday |  |  |  | 312 |
| Friday |  |  | 48 |  |

1. Decrease her morning NPH to 0.5 units because she is frequently low at suppertime.
2. Increase her evening regular insulin to 1.5 units because her bedtime blood sugar is higher than target
3. Decrease her suppertime regular insulin because his bedtime blood sugars are high
4. Advise the mother to give her evening regular and NPH insulin closer to when she will eat supper.

Problem 18:

A 13 year old boy comes in for a check up. He has no complaints and no concerns. Mom says that he eats well at mealtimes, which usually consists of rice and vegetables. His weight for height is at the 44^th^ percentile. His HbA1c is 4.8%. How do you assess his blood sugars?

|  | **Breakfast** | **Lunch** | **Supper** | **Bedtime** |
| --- | --- | --- | --- | --- |
| Monday | 82 |  | 45 – felt hungry |  |
| Tuesday | 54 – no symptoms |  |  |  |
| Wednesday |  |  |  | 62 – no symptoms |
| Thursday |  | 37 – felt shaky |  |  |
| Friday |  |  |  |  |

1. Decrease all his insulin doses so that he is within target most of the time, so that he can regain awareness of his hypoglycemia over time.
2. Decrease his morning NPH and regular doses because he had hypoglycemia symptoms at mid-day and supper.
3. Decrease his suppertime insulin because he was low in the morning
4. Remind him to eat sufficient carbohydrates for his insulin doses.

Problem 19:

A 22 year old diabetes patient comes in with the following numbers. He takes:

|  | 9 AM | 5 PM |
| --- | --- | --- |
| Regular | 8 | 6 |
| NPH | 18 | 15 |

He eats breakfast at 9 AM, lunch at 12 PM and supper at 5 PM.

|  | **Breakfast** | **Lunch** | **Supper** | **Bedtime** |
| --- | --- | --- | --- | --- |
| Monday | 123 |  |  |  |
| Tuesday |  | 191 |  |  |
| Wednesday |  | 203 | 137 |  |
| Thursday |  |  |  |  |
| Friday |  |  | 98 | 154 |

1. He needs to increase his insulin at breakfast time because he is high by lunch time.
2. He needs to wait 30 minutes after his shot to eat, so that his regular insulin peaks with the blood sugar from his food**.**
3. He should increase his NPH to help correct his highs at lunch time.
4. He should increase both his regular and NPH insulin.

Problem 20

12 year old diabetic, doses as below:

|  | Morning | Evening |
| --- | --- | --- |
| Regular | 7 | 5 |
| NPH | 14 | 9 |

|  | **Breakfast** | **Lunch** | **Supper** | **Bedtime** |
| --- | --- | --- | --- | --- |
| Monday | 115 |  |  |  |
| Tuesday |  | 191 | 345 |  |
| Wednesday |  |  | 203 | 276 |
| Thursday | 170 |  |  | 389 |
| Friday | 141 |  |  |  |

1. increase morning NPH to 16 units, increase evening regular insulin to 6 units
2. Increase morning regular to 8 units, decrease morning NPH to 13 units
3. Increase morning regular to 8 units, increase evening NPH to 10 units
4. Increase morning regular to 8 units, increase evening regular to 6 units.

Problem 21

17 year old diabetic, doses as below:

|  | Morning | Evening |
| --- | --- | --- |
| Regular | 13 | 8 |
| NPH | 20 | 14 |

|  | **Breakfast** | **Lunch** | **Supper** | **Bedtime** |
| --- | --- | --- | --- | --- |
| Monday |  |  | 128 | 67 |
| Tuesday | 191 |  |  |  |
| Wednesday | 98 | 203 |  |  |
| Thursday |  | 256 | 89 |  |
| Friday |  |  | 137 | 68 |

1. Increase morning NPH to 22 units, decrease evening NPH to 13 units
2. Increase morning NPH to 22 units, decrease evening NPH to 12 units
3. Increase morning regular to 14 units, decrease evening regular to 5 units
4. Increase morning regular to 14 units, decrease evening regular to 7 units

Problem 21

6 year old diabetic, doses as below:

|  | Morning | Evening |
| --- | --- | --- |
| Regular | 1 | 1 |
| NPH | 4 | 2 |

|  | **Breakfast** | **Lunch** | **Supper** | **Bedtime** |
| --- | --- | --- | --- | --- |
| Monday |  |  |  | 124 |
| Tuesday | 318 | 138 |  |  |
| Wednesday |  | 203 | 91 | 96 |
| Thursday | 473 | 256 |  |  |
| Friday |  |  |  | 147 |

1. More information is needed – add more bedtime checks
2. More information is needed – add overnight checks
3. Increase morning regular to 1.5 units, increase evening NPH to 2.5 units
4. Increase evening regular insulin to 1.5 units, decrease evening NPH to 1.5 units

Problem 22

10 year old diabetic, doses as below:

|  | Morning | Evening |
| --- | --- | --- |
| Regular | 3 | 3 |
| NPH | 10 | 5 |

|  | **Breakfast** | **Lunch** | **Supper** | **Bedtime** |
| --- | --- | --- | --- | --- |
| Monday |  |  | 146 | 367 |
| Tuesday | 254 | 132 |  |  |
| Wednesday |  | 148 | 78 | 164 |
| Thursday | 137 | 89 |  |  |
| Friday |  |  | 72 | 155 |

1. Increase evening NPH to 6 units
2. Decrease morning NPH to 9 units
3. Decrease morning regular insulin to 2.5 units
4. Increase evening regular to 4 units.

Problem 23

17 year old diabetic is taking the following doses:

40 units of 70/30 insulin at breakfast, 20 units of 70/30 insulin at suppertime

|  | **Breakfast** | **Lunch** | **Supper** | **Bedtime** |
| --- | --- | --- | --- | --- |
| Monday |  |  | 48 | 156 |
| Tuesday | 123 |  |  |  |
| Wednesday |  | 216 | 55 |  |
| Thursday |  |  |  |  |
| Friday | 137 | 273 |  |  |

1. Switch patient to a combination of NPH and regular insulin – give 25 units of NPH and 14 units of regular in the morning, 14 units of NPH and 6 units of regular in the evening.
2. Decrease morning 70/30 to 36 units
3. Increase morning 70/30 to 42 units
4. Switch patient to a combination of NPH and regular insulin – give 28 units of NPH and 14 units of regular in the morning, 14 units of NPH and 6 units of regular in the evening.

Problem 24

21 year old diabetic is taking the following doses:

35 units of 70/30 insulin (70% NPH, 30% regular) at breakfast, 18 units of 70/30 insulin at suppertime

|  | **Breakfast** | **Lunch** | **Supper** | **Bedtime** |
| --- | --- | --- | --- | --- |
| Monday | 141 |  |  |  |
| Tuesday | 123 | 83 | 151 |  |
| Wednesday |  |  |  | 315 |
| Thursday | 215 | 69 |  |  |
| Friday |  | 78 | 73 | 256 |

1. Decrease evening 70/30 to 16 units
2. Decrease morning 70/30 to 33 units
3. Switch patient to a combination of NPH and regular insulin – give 10.5 units of regular and 24.5 units of NPH at breakfast; give 5.5 units of regular and 11 units of NPH in the evening.
4. Switch patient to a combination of NPH and regular insulin – give 10.5 units of regular and 24.5 units of NPH in the morning; give 7 units of regular and 12.5 units of NPH in the evening.

Problem 25

15 year old diabetic, doses as below:

|  | Morning | Evening |
| --- | --- | --- |
| Regular | 16 | 10 |
| NPH | 30 | 20 |

|  | **Breakfast** | **Lunch** | **Supper** | **Bedtime** |
| --- | --- | --- | --- | --- |
| Monday |  |  | 57 | 316 |
| Tuesday | 89 |  |  |  |
| Wednesday |  |  |  |  |
| Thursday | 91 | 153 | 67 |  |
| Friday |  |  |  |  |

1. No clear pattern; no changes
2. Increase the morning regular dose to 18 units
3. Decrease the morning NPH to 27 units
4. Increase the evening regular dose to 11 units.

Problem 26

16 year old diabetic, doses as below:

|  | Morning | Evening |
| --- | --- | --- |
| Regular | 12 | 8 |
| NPH | 24 | 10 |

|  | **Breakfast** | **Lunch** | **Supper** | **Bedtime** |
| --- | --- | --- | --- | --- |
| Monday |  | 94 | 281 | 162 |
| Tuesday | 89 | 46 |  |  |
| Wednesday |  |  |  |  |
| Thursday |  | 67 | 338 |  |
| Friday |  | 71 | 274 |  |

1. In the morning, decrease regular insulin and increase NPH.
2. In the morning, increase regular insulin and decrease NPH.
3. In the evening, decrease regular insulin and increase NPH.
4. In the evening, increase regular insulin and decrease NPH.

Problem 27

25 year old diabetic, doses as below:

|  | Morning | Evening |
| --- | --- | --- |
| Regular | 24 | 12 |
| NPH | 40 | 24 |

|  | **Breakfast** | **Lunch** | **Supper** | **Bedtime** |
| --- | --- | --- | --- | --- |
| Monday | 151 | 90 | 180 |  |
| Tuesday |  |  |  | 95 |
| Wednesday | 132 |  | 134 | 216 |
| Thursday | 87 | 194 | 153 |  |
| Friday |  |  | 127 | 102 |

1. No clear pattern; no changes
2. Increase the morning NPH to 43 units
3. Increase the morning regular insulin to 26 units
4. Increase the evening regular insulin to 14 units.

Problem 28

23 year old female, doses as below:

|  | Morning | Evening |
| --- | --- | --- |
| Regular | 18 | 11 |
| NPH | 33 | 23 |

|  | **Breakfast** | **Lunch** | **Supper** | **Bedtime** |
| --- | --- | --- | --- | --- |
| Monday | 120 | 210 | 364 |  |
| Tuesday |  |  | 257 | 318 |
| Wednesday | 93 |  |  |  |
| Thursday |  |  | 161 | 273 |
| Friday | 146 | 172 | 281 |  |

1. Increase all insulin doses by 1-2 units
2. Increase morning regular and NPH by 1-2 units; increase evening regular and decrease evening NPH.
3. Increase morning regular and NPH; decrease evening regular and increase evening NPH.
4. Decrease morning regular and increase morning NPH; leave evening doses unchanged.

Problem 29

13 year old female, doses as below:

|  | Morning | Evening |
| --- | --- | --- |
| Regular | 9 | 5 |
| NPH | 14 | 8 |

|  | **Breakfast** | **Lunch** | **Supper** | **Bedtime** |
| --- | --- | --- | --- | --- |
| Monday | 271 |  |  | 141 |
| Tuesday |  |  |  | 101 |
| Wednesday | 324 |  |  |  |
| Thursday |  |  |  | 180 |
| Friday | 93 |  |  |  |

1. Increase morning NPH to 16 units
2. Increase evening regular to 6 units.
3. Need more information – check blood sugar overnight.
4. Increase evening NPH to 9 units.

Problem 30

17 year old male, doses as below:

|  | Morning | Evening |
| --- | --- | --- |
| Regular | 5 | 4 |
| NPH | 17 | 9 |

|  | **Breakfast** | **Lunch** | **Supper** | **Bedtime** |
| --- | --- | --- | --- | --- |
| Monday | 180 | 156 |  |  |
| Tuesday |  | 83 | 134 | 94 |
| Wednesday | 257 |  |  |  |
| Thursday |  |  |  | 243 |
| Friday | 127 | 120 | 141 |  |

1. Increase evening regular insulin
2. Increase evening NPH insulin.
3. Both A and B.
4. Need more information – check glucoses overnight.
5. Both A and D.

Problem 31

16 year old male, doses as below:

|  | Morning | Evening |
| --- | --- | --- |
| Regular | 8 | 4 |
| NPH | 16 | 8 |

|  | **Breakfast** | **Lunch** | **Supper** | **Bedtime** |
| --- | --- | --- | --- | --- |
| Monday |  | 200 | 137 | 142 |
| Tuesday | 109 |  |  |  |
| Wednesday |  |  | 163 | 132 |
| Thursday | 134 | 361 | 108 |  |
| Friday |  | 162 | 98 | 105 |

1. Increase morning NPH
2. Increase morning regular insulin.
3. Decrease evening regular insulin.
4. No consistent pattern – no changes for now.

Problem 32

13 year old female, doses as below:

|  | Morning | Evening |
| --- | --- | --- |
| Regular | 9 | 4 |
| NPH | 16 | 8 |

|  | **Breakfast** | **Lunch** | **Supper** | **Bedtime** |
| --- | --- | --- | --- | --- |
| Monday | 134 | 56 | 379 |  |
| Tuesday |  | 74 | 123 | 136 |
| Wednesday |  |  |  | 151 |
| Thursday | 141 | 42 | 286 |  |
| Friday |  |  | 89 |  |

1. Decrease morning regular insulin
2. Increase morning NPH
3. Both A and B.
4. No clear pattern – make no changes.

Problem 33

21 year old male, doses as below:

|  | Morning | Evening |
| --- | --- | --- |
| Regular | 10 | 6 |
| NPH | 25 | 13 |

|  | **Breakfast** | **Lunch** | **Supper** | **Bedtime** |
| --- | --- | --- | --- | --- |
| Monday | 67 | 187 |  |  |
| Tuesday |  | 97 | 105 | 89 |
| Wednesday | 59 |  |  | 167 |
| Thursday | 71 | 89 |  |  |
| Friday |  |  |  |  |

1. Decrease evening regular insulin
2. Increase morning regular insulin
3. Decrease evening NPH
4. No clear pattern – no changes for now

Problem 34

18 year old male, doses as below:

|  | Morning | Evening |
| --- | --- | --- |
| Regular | 9 | 6 |
| NPH | 21 | 12 |

|  | **Breakfast** | **Lunch** | **Supper** | **Bedtime** |
| --- | --- | --- | --- | --- |
| Monday | 282 | 72 |  |  |
| Tuesday |  |  | 143 | 162 |
| Wednesday | 89 |  |  |  |
| Thursday |  | 65 | 178 | 91 |
| Friday |  |  |  |  |

1. Increase evening NPH
2. Decrease morning regular.
3. Both A and B.
4. Increase morning NPH.

Problem 35

12 year old female, doses as below:

|  | Morning | Evening |
| --- | --- | --- |
| Regular | 5 | 3 |
| NPH | 11 | 6 |

|  | **Breakfast** | **Lunch** | **Supper** | **Bedtime** |
| --- | --- | --- | --- | --- |
| Monday |  |  | 127 | 89 |
| Tuesday | 136 | 81 |  |  |
| Wednesday |  |  |  | 93 |
| Thursday | 141 | 121 |  |  |
| Friday |  | 192 | 154 |  |

1. Increase morning NPH
2. Decrease evening regular insulin.
3. Increase morning regular insulin.
4. Make no changes – most blood sugars are in an acceptable range.

Problem 36

15 year old female 24 units of 70/30 in the morning and 14 units of 70/30 in the evening:

|  | **Breakfast** | **Lunch** | **Supper** | **Bedtime** | **Overnight** |
| --- | --- | --- | --- | --- | --- |
| Monday |  | 89 | 121 | 191 |  |
| Tuesday | 251 |  |  | 163 | 189 |
| Wednesday | 226 | 137 |  |  |  |
| Thursday |  | 123 | 89 |  |  |
| Friday |  |  | 75 | 163 | 287 |

1. Increase evening 70/30 to 16 units
2. Switch to NPH and regular – give 17 units of NPH and 7 units of regular during the day, 10 units of NPH and 4 units of regular in the evening
3. Switch to NPH and regular – give 17 units of NPH and 7 units of regular insulin during the day, 12 units of NPH and 3 units of regular in the evening.
4. Switch to NPH and regular – give 19 units of NPH and 8 units of regular in the morning, 10 units of NPH and 4 units of regular in the evening.

Problem 37

20 year old male, doses as below:

|  | Morning | Evening |
| --- | --- | --- |
| Regular | 7 | 4 |
| NPH | 17 | 10 |

|  | **Breakfast** | **Lunch** | **Supper** | **Bedtime** | **Overnight** |
| --- | --- | --- | --- | --- | --- |
| Monday | 251 | 135 | 69 | 136 |  |
| Tuesday |  |  |  | 121 | 62 |
| Wednesday | 301 |  |  |  |  |
| Thursday | 296 |  |  |  |  |
| Friday |  | 98 | 65 | 102 |  |

1. Increase evening NPH.
2. Decrease morning NPH.
3. Decrease evening NPH.
4. A and B
5. B and C

Problem 38

19 year old male, doses as below:

|  | Morning | Evening |
| --- | --- | --- |
| Regular | 12 | 6 |
| NPH | 25 | 12 |

|  | **Breakfast** | **Lunch** | **Supper** | **Bedtime** |
| --- | --- | --- | --- | --- |
| Monday | 167 | 78 | 124 |  |
| Tuesday |  |  |  | 136 |
| Wednesday | 89 | 191 |  |  |
| Thursday |  | 42 | 376 |  |
| Friday |  |  | 89 |  |

1. Decrease evening NPH
2. Increase morning regular insulin
3. Increase morning NPH
4. No clear pattern – make no changes.

Problem 39

9 year old male, doses as below:

|  | Morning | Evening |
| --- | --- | --- |
| Regular | 5 | 3 |
| NPH | 12 | 6 |

|  | **Breakfast** | **Lunch** | **Supper** | **Bedtime** |
| --- | --- | --- | --- | --- |
| Monday |  |  |  | 145 |
| Tuesday | 378 | 216 | 151 |  |
| Wednesday |  | 149 | 89 | 91 |
| Thursday | 268 |  |  |  |
| Friday | 312 | 138 | 143 |  |

1. Increase morning NPH
2. Increase morning regular.
3. Increase evening NPH.
4. Obtain overnight glucoses before making changes

Problem 40!!

25 year old male, doses as below:

|  | Morning | Evening |
| --- | --- | --- |
| Regular | 9 | 5 |
| NPH | 19 | 10 |

|  | **Breakfast** | **Lunch** | **Supper** | **Bedtime** |
| --- | --- | --- | --- | --- |
| Monday | 123 | 281 | 164 |  |
| Tuesday |  | 196 | 84 | 136 |
| Wednesday | 102 |  |  |  |
| Thursday |  |  |  | 112 |
| Friday | 89 | 213 | 76 |  |

1. Increase morning regular insulin
2. Decrease evening NPH.
3. Increase morning NPH.
4. No clear pattern – make no changes
